# C-reactive protein for late-onset sepsis diagnosis in very low birth weight infants

**DOI:** 10.1186/s12887-018-1002-5

**Published:** 2018-01-30

**Authors:** Marc Beltempo, Isabelle Viel-Thériault, Roseline Thibeault, Anne-Sophie Julien, Bruno Piedboeuf

**Affiliations:** 1 McGill University Health Centre, Montreal, QC, Canada; 2 Département de pédiatrie, Centre Mère-Enfant Soleil du CHU de Québec, Université Laval, 2705 Boulevard Laurier, QC, Québec G1V 4G2 Canada; 3 Centre de recherche du CHU de Québec, Université Laval, QC, Québec Canada

**Keywords:** C-reactive protein, Late-onset sepsis, Neonatology, Very low birth weight

## Abstract

**Background:**

Late-onset sepsis in very low birth weight (VLBW) infants is a diagnostic challenge. We aimed to evaluate the diagnostic utility of the C-Reactive protein (CRP) and the complete blood count (CBC) for late-onset sepsis in VLBW infants.

**Methods:**

In a 5-year retrospective cohort of 416 VLBW infants born at less than 1500 g, there were 590 separate late-onset sepsis evaluations. CRP and CBC were drawn at time of initial blood culture (T0), at 16–24 h (T24) and 40–48 h (T48) after. The positive cut-off values for abnormal values were the following: CRP ≥10 mg/L and CBC with at least one anomaly, including white blood cell count < 5000/mm^3^, immature neutrophil/total neutrophil ratio > 0.10, or platelet count < 100,000/uL. Sensitivity and specificity for predicting late-onset sepsis were calculated for each laboratory test and their combinations. Receiver operating characteristics curves were obtained for each test and for the absolute change from T0 to T24 in the laboratory value of CRP, white blood cell count and immature neutrophil/total neutrophil.

**Results:**

At T0, combining the CBC and the CRP had the highest sensitivity of 66% (95% confidence interval [CI], 58–73) compared to both individual tests for predicting late onset sepsis. At T24, CRP’s sensitivity was 84% (95% CI, 78–89) and was statistically higher than the CBC’s 59% (95% CI, 51–67). The combination of CBC at T0 and CRP at T24 offered the greatest sensitivity of 88% (95% CI, 82–92) and negative predictive value 93% (95% CI, 89–96), with fewer samples, compared to any other combination of tests. The area under the curve for the change in the white blood cell count from T0 to T24 was 0.82.

**Conclusion:**

At initial sepsis evaluation (T0), both CBC and CRP should be performed to increase sensitivity. A highly negative predictive value is reachable with only two tests: a CBC at T0 and a CRP a T24.

## Background

Late-onset sepsis represents significant morbidity and mortality in the neonatal intensive care unit (NICU) as it occurs in 16 to 25% of very low birth weight (VLBW) infants (birth weight < 1500 g) [1–7] It has also been associated with prolonged hospital stay [3, 4, 8] and long-term neurodevelopmental impairment [3, 6, 9]. The diagnosis of late-onset sepsis in VLBW infants is difficult due to subtle and non-specific clinical signs [4]. This is why many studies have proposed the use of laboratory markers as adjunctive diagnostic tools. C-reactive protein (CRP) is a well-described acute phase reactant that is synthesized by the liver in response to pro-inflammatory cytokines 4 to 6 h after an initial trigger, like infection or tissue injury. It significantly rises 10 to 12 h and peaks 24 to 48 h after the initial insult [10–12].

Many studies have assessed the use of CRP for the diagnosis of early-onset sepsis in term and late-preterm infants [13–15]. In these populations, two CRP values of < 10 mg/L have a negative predictive value of 93 to 97% [2, 12, 16, 17]. Preterm infants born at ≤32 weeks’ gestational age have a comparable CRP response in early-onset bacterial infections compared to infants born > 32 weeks’ gestational age [14]. However, less is known about the CRP response of VLBW infants in late-onset sepsis. Indeed, coagulase-negative *staphylococci (*CoNS*)* are the most common causative pathogen of late-onset sepsis among VLBW infants in Canadian NICUs [18] and previous studies suggest that CoNS are associated with lower levels of inflammation compared to other bacteria [19, 20]. Also, little is known about the meaning significance of the variation of CRP values between two time points.

The complete blood count (CBC) is used by 99% of the clinicians as part of their initial sepsis evaluation [7]. However, no single marker possesses adequate sensitivity to rule out late-onset sepsis in VLBW infants [7]. CBC parameters previously associated with late-onset sepsis include a total white blood cell count (WBC) < 5000/mm^3^, an immature neutrophil/total neutrophil (I/T) ratio > 0.10 and a platelet count lower than < 100,000/uL [1]. The use of CBC in combination with CRP for late-onset sepsis evaluation in VLBW infants could potentially be more sensitive than each individual test. It is also possible that the variation in time (from T0 to T24) of these tests could be clinically useful even when the absolute test results are below the cut-off of abnormal values.

We conducted a retrospective cohort study to evaluate the use of CRP in the diagnosis of late-onset sepsis in VLBW infants. Specifically, we aimed (1) to assess sensitivity, specificity, positive and negative predictive values of CRP compared to the CBC and (2) to identify the combination of tests that offers the highest sensitivity for the diagnosis of late-onset sepsis. Additionally, we assessed the predictive value of the variations from 0 to 24 h after initial evaluation of the CRP, the WBC and the I/T ratio.

## Methods

### Study population

This retrospective study was performed at the CHU de Quebec – Université Laval NICU (Quebec, Canada), between March 2008 and April 2013. The study was approved by the Institutional Research and Ethics Board. The dataset analyzed for the current study is available from the corresponding author upon reasonable request. All neonates with a birth weight < 1500 g and more than three days old at initial late-onset sepsis evaluation were included.

### Case definition

Late-onset sepsis evaluation was defined as a symptomatic patient having a blood culture drawn. Common clinical indications for late-onset sepsis evaluations included increased apnea episodes, temperature instability, feeding intolerance, lethargy and hypotonia. Infants had proven late-onset sepsis if the blood culture or cerebrospinal fluid culture drawn as part of the initial work-up was positive for bacterial pathogens. There was no mandatory requirement for two distinct blood cultures since it is not the routine practice for VLBW infants in this NICU. CoNS were considered as pathogens if the infant was symptomatic and treated with antibiotics for more than two days with clinical improvement. We excluded known contaminants such as *Corynebacterium* and unidentified organisms. Late-onset sepsis evaluations occurring within 14 days from the initial evaluation in a same patient were excluded to ensure they were different episodes as opposed to blood culture controls drawn during treatment. Episodes of sepsis occurring more than 14 days apart were included as separate episodes.

### Data collection

The hospital clinical database *Med-Echo*, a national validated medico-administrative database was used to collect patient demographics [21]. The local infectious disease database *TDR* was used to collect dates of blood cultures. Individual patient laboratory and clinical data were collected using electronic medical charts. Time of initial evaluation (T0) was defined as the of the initial blood culture. CBC and CRP values were collected at T0, at 16–24 h (T24) and 40–48 h (T48) after.

### Laboratory cut-offs

CRP levels were determined using Vitros CRP slide method (Vitros 250 Chemistry System, Ortho-Clinical Diagnostic, Johnson and Johnson). The CRP was considered positive if the laboratory value was ≥10 mg/L. The CBC was considered abnormal if any of the following was present: white blood cell count (WBC) < 5000/mm^3^, I/T ratio > 0.10 or platelet count < 100,000/uL.

### Data analysis

Continuous variables with normal distributions are presented with the mean and standard deviation, while continuous variables with non-normal distributions are presented with the median and interquartile range (IQR). Qualitative variables are presented with frequency and percentage. Analysis of variance, estimated with generalized estimated equations (GEE), was used to test for differences in median CRP results at T0, T24 and T48 between the different bacterial pathogens. GEE were used to calculate sensitivity, specificity, positive and negative predictive values with 95% confidence intervals (CI) for predicting late-onset sepsis for the CRP and CBC at T0, T24 and T48 as well as for different combinations of these tests. Logistic regressions were used to compare sensitivity and specificity between the CRP, the CBC and different combinations. Multiple comparisons were corrected using Bonferroni method. Patients with rapidly resolving clinical symptoms with a normal CBC and CRP at T0 might not have had repeat testing done at T24 and T48 and patients with abnormal results (CBC or CRP) at T0 may not have had repeated tests since the early markers were already positive. Both situations could lead to a selection bias by analyzing the available values at those time points because using only available data may change the prevalence of the disease and affect the diagnostic accuracy of the blood tests [22]. Consequently, last observation carried forward (LOCF) imputation technique was used for missing values when data at previous times was available to limit a potential selection bias.

Receiver-operating characteristic (ROC) curves were used to assess the diagnostic accuracy of each tesst through the area under the curve (AUC) estimates. Specifically, performance of the following markers was analyzed: the CRP done 24 h after initial workup and the absolute difference in its values from T0 to T24, the white blood cell count and the I/T ratio. *P* values < 0.05 were considered significant. All statistical analyses were performed with SAS, version 9.3 (SAS Institute Inc., Cary, NC), while ROC curves were produced by IBM SPSS Statistics for Windows, version 22 (IBM Corp., Armonk, NY).

## Results

### Patient characteristics and types of infections

During the 5-year period, 1090 blood cultures were performed in 416 eligible VLBW infants. A total of 590 distinct late-onset sepsis evaluations met the inclusion criteria. Of the 500 excluded blood cultures, 481 represented repeated blood cultures done within 14 days of the initial late-onset sepsis evaluation, 9 were probable contaminants and 10 had no available associated CRP data at all three time points. The demographic characteristics of the patients included are detailed in Table 1. In total, 162 (27%) evaluations were culture proven late-onset sepsis, all had a least one positive blood culture. CoNS were isolated in 83% of the episodes of infection, and the remainder were caused by other gram-positive bacteria (9%), gram-negative bacteria (7%) and fungi (1%). Among the 162 blood culture-proven late-onset sepsis episodes, 3 had meningitis (1 fungal and 2 bacterial) and 6 had a urinary tract infection. There were no cases of meningitis with a negative blood culture.

**Table 1:** Demographic characteristics of the 416 patients included

Patients characteristics	Value
Gestational age (weeks), mean ± SD	27.9 ± 2.4
Birth weight (g), mean ± SD	1024.8 ± 258.1
Sex	
Male, *n* (%)	231 (56)
Female, *n* (%)	185 (44)
Twin pregnancy	
Yes, *n* (%)	124 (30)
No, *n* (%)	292 (70)
C-section delivery	
Yes, n (%)	297 (72)
No, *n* (%)	119 (28)
5 min Apgar < 8	
Yes, *n* (%)	145 (35)
No, *n* (%)	271 (65)
Death	
Yes, *n* (%)	25 (6)
No, *n* (%)	391 (94)
≥ 1 positive blood culture	
Yes, *n* (%)	126 (30)
No, *n* (%)	290 (70)
Age at first sepsis evaluation (days), mean ± SD	15.0 ± 12.8

### CRP increase

After LOCF imputation, there were 575, 583 and 586 available CR*P* values at T0, T24 and T48 respectively. CRP peaked at 24 h irrespective of the causative pathogen. At T24, the median CRP values were 38 mg/L for gram-positive bacterial infections other than CoNS, 40 mg/L for CoNS infections and 90 mg/L for gram-negative bacterial infections (Table 2). At T0, T24 and T48, all comparisons of CRP values between pathogens were not statistically significantly different (all P values > 0.80). There were 116 (48%) false positive CRP tests at T24 (CRP > 10 and no late-onset sepsis): 14 were treated for necrotizing enterocolitis, 8 had tests taken when the infant was in a postoperative period and 38 had suspected ventilator-associated pneumonia. At T24, there were 25 (8%) false negative CRP tests (CRP ≤10 with late-onset sepsis) for which the causative organisms are listed in Table 3.

**Table 2:** Pathogens isolated in blood cultures and their mean serial CRP values

Organisms (*N*)	CRP at T0 (mg/L), median, [IQR]	CRP at T24 (mg/L), median, [IQR]	CRP at T48 (mg/L), median, [IQR]
Coagulase-negative *staphylococci* (135)	10 [3–23]	40 [15–58]	19 [9–34]
Non-CoNS gram-positive bacteria (15)	8.0 [2–23]	38 [17–97]	38 [10–97]
Gram-negative bacteria (11)	32 [2–56]	90 [15–140]	93 [13–90]
Fungi (1)	6 [6–6]	6 [6–6]	6 [6–6]

Abbreviations: *CoNS* Coagulase-negative *staphylococci, IQR* Interquartile range

Note: At T0, T24 and T48, all comparisons of CRP values between pathogens were not statistically significantly different (all *P* values > 0.80 obtained by generalized estimated equations and adjusted with Bonferonni correction for multiples testing)

**Table 3:** Organisms isolated from positive blood cultures in patients with confirmed infections and CRP < 10 mg/L or ≥10 mg/L at T24

Organisms (*N*)	CRP < 10 mg/L (*N* = 25)	CRP ≥ 10 mg/L (*N* = 137)
Coagulase-negative staphylococci	Coagulase-negative staphylococci (19)	Coagulase-negative staphylococci (116)
Non-CoNS gram-positive bacteria	*Staphylococcus aureus* (1)	Staphylococcus aureus (6)
	Enterococcus faecalis (1)	Enterococcus faecalis (2)
	*Streptococcus agalactiae* (1)	Streptococcus agalactiae (4)
Gram-negative bacteria	*Enterobacter cloacae* (1)	Enterobacter cloacae (3)
	*Klebsiella pneumoniae* (1)	Klebsiella pneumoniae (4)
		Escherchia coli (2)
Fungi	*Candida lusitaniae* (1)	

### Sensitivity, specificity, positive predictive and negative predictive values of CRP and CBC

The sensitivity, specificity, positive and negative predictive values of the CRP, CBC and their combinations were calculated at each time point and compared (Table 4). At initial sepsis work-up (T0), combining the CBC and CRP offered the highest sensitivity for late-onset sepsis diagnosis (65%) which was statistically superior to CRP (49%, *p* < 0.001) and CBC (49%, p < 0.001) alone. At T24, the sensitivity of the CRP increased, and was not statistically significantly different than when combined with the CBC (84% vs 87%, *p* = 0.36). At T24, the sensitivities of the individual components of the CBC were 50% for the I/T ratio, 4% for leukopenia and 12% for thrombocytopenia. Compared to its performance at T24, the sensitivity of the CRP at T48 decreased (84% vs 73%, *p* = 0.08). At T48, the sensitivity of the combined CBC and CRP was similar to the individual CRP (76% vs 73%, *p* = 0.35). The sensitivity of the CBC at T48 significantly decreased compared to T24 (21% vs 59%, *p* < 0.001).

**Table 4:** Sensitivity, specificity, positive and negative predictive values of tests at T0, T24 h and T48h^a^

Test	Sensitivity	Specificity	PPV	NPV
T0				
CRP	49% (41–56)	76% (72–80)	43% (37–50)	79% (75–83)
CBC	49% (41–57)	83% (79–86)	52% (44–60)	81% (77–84)
CBC + CRP	65% (57–72)†	66% (61–70)†	42% (36–48)	83% (79–87)
T24 H				
CRP	84% (78–89)	70% (66–75)	52% (46–58)	92% (89–95)
CBC	59% (51–67)†	79% (75–83)	53% (46–60)	84% (80–87)
CBC + CRP	87% (80–91)	60% (55–64)†	45% (40–51)	92% (88–95)
T 48H				
CRP	73% (66–79)	79% (74–82)	57% (50–63)	88% (85–91)
CBC	21% (15–28)†	92% (88–94)†	50% (39–62)	75% (71–79)
CBC + CRP	76% (69–82)	74% (69–78)	53% (46–59)	89% (85–92)

^a^Parenthesis indicate 95% confidence interval

†Sensitivity or specificity significantly different (*p* < 0.05) compared to the CRP taken at the same time

### Optimal test combinations

Table 5 presents multiple test combinations at different times. The maximum sensitivity and negative predictive values were 88% and 93% respectively, and could be obtained by performing only a CBC at T0 with a CRP at T24. Also, the sensitivity obtained with three consecutive CRP measurements at T0, T24 and T48 was 86% which was not superior to the sensitivity of a single CRP at T24 (*p* = 0.93).

**Table 5:** Sensitivity, specificity, positive and negative predictive values of selected combined tests^a^

Tests combinations			Sensitivity	Specificity	PPV	NPV
T0	T24	T48				
CBC	CRP		88% (82–92)	60% (55–65)	46% (41–52)	93% (89–96)
CBC + CRP	CRP		88% (82–92)	60% (55–65)	46% (41–52)	93% (89–96)
CBC + CRP	CBC + CRP		88% (82–92)	56% (51–61)	44% (38–49)	92% (88–95)
CBC + CRP			65% (57–72)	66% (61–70)	42% (36–48)	83% (79–87)
	CBC + CRP		87% (81–92)	59% (54–64)	45% (40–51)	93% (89–95)
CRP	CBC		74% (67–81)	62% (57–67)	43% (37–49)	86% (82–90)
CRP	CRP		84% (78–89)	70% (65–74)	52% (46–58)	92% (88–95)
CRP	CRP	CRP	86% (79–90)	69% (64–73)	52% (46–58)	93% (89–95)

^a^Parenthesis indicate 95% confidence interval

### Absolute variations of CRP, WBC and I/T ration from T0 to T24

**Fig. 1:**
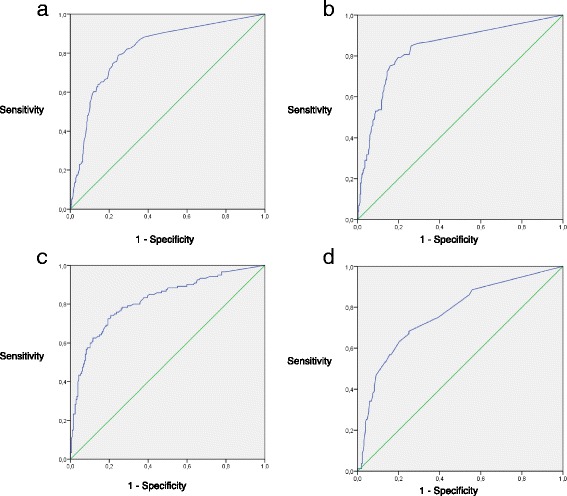
Receiver operative characteristic (ROC) curves of different tests. **a**. CRP at T24. AUC = 0.82 (95% CI, 0.78–0.86). **b**. Absolute difference in the CR*P* values obtained at T0 and T24. AUC, 0.84 (95% CI, 0.79–0.88). **c**. Absolute difference in the white blood cell count at T0 and T24. AUC 0.82 (95% CI, 0.77–0.87). **d**. Absolute difference in the I/T ratio at T0 and T24. AUC 0.77 (95% CI, 0.70–0.82)

ROC curves and the corresponding AUC of different tests are presented in Fig. 1. The AUC of a single CRP measured at T24 was 0.82 and was not statistically significantly different than the variations in CRP, white blood cell count and I/T ratio (all *P* values > 0.75).

## Discussion

### Diagnostic accuracy of CRP

This study focused on the use of CRP as an adjunctive diagnostic tool in the evaluation of VLBW infants with suspected late-onset sepsis. The sensitivity of the CRP at initial evaluation (T0) was low (49%), which correlates with previous studies in other neonatal populations [12, 15]. Nevertheless, at 24 h, CRP had a better sensitivity of 84% for late-onset sepsis and a high negative predictive value of 92%. This is similar to previously published results in in cohorts of infants born at different gestational ages [12, 15]. Serial measurement of CRP at T0, T24 and T48 was associated with a 93% negative predictive value, which is lower than what other prospective studies have reported (98%) [16]. However, those studies included smaller numbers of VLBW infants and had a higher prevalence of gram-negative bacterial sepsis.

### CRP increase and CoNS sepsis

We report a high rate of CoNS sepsis compared to previous studies on CRP [14, 16, 20]. However, these rates are comparable to the incidence of CoNS infections in the Canadian Neonatal Network (> 70% of late-onset sepsis are caused by CoNS) [18]. This is likely attributable to variations in local epidemiology combined with care practices. Indeed, the rate of fungal infections was also very low in our cohort and similar to the Canadian average (incidence < 2% in VLBW infants) [18]. The fact that we did not mandate two cultures for the diagnosis of sepsis may have contributed to a higher prevalence of contaminants. This may have increased the false positive rate in our cohort, but would have little effect on the negative predictive value of CRP.

Previous studies concluded that CoNS might induce a less sustained inflammatory response than gram-negative bacterial sepsis [13, 14]. However, our findings do not suggest a statistically significant difference in peak CRP’s values in infants with CoNS compared to other pathogens, however this is not statistically significant given the small number of gram-negative infections. Likewise, 46% of the CoNS sepsis included in our cohort were associated with a CRP value of at least 50 mg/L suggesting that the inflammatory potential of these organisms is present.

### Diagnostic accuracy of the CBC

We found that a CBC obtained at T24 has a low sensitivity (59%) for the diagnosis of late-onset sepsis in VLBW infants. The I/T ratio was the main contributor to the diagnostic accuracy of the CBC at T24 (sensitivity 50%). This is in keeping to what has been reported in more mature newborns. Indeed, Hornik et al. found that the sensitivity of the CBC in infants born < 34 weeks gestational age was 55% [7]. However, they used a different cut-off for the I/T ratio (> 0.2). They noted a higher I/T ratio in gram-negative infections. The low incidence of gram-negative infections in our cohort might explain why a lower threshold yielded similar results. Moreover, as a late-onset physiologic neutropenia frequently occurs in VLBW infants, their I/T ratio might be less accurate [23]. Consequently, using a lower positivity threshold may be appropriate in order to increase its sensitivity as a screening tool in this specific population. A platelet count < 100,000/uL at T24 had a low sensitivity (12%) for predicting late-onset sepsis. This is similar to previous studies that found the platelet count has a low discriminative performance (AUC 0.60) for diagnosing sepsis in preterm infants [7]. The variation in platelet count based on gestational age and postnatal age may require the use of age specific cut-offs [24]. Also, platelet parameters like mean platelet volume and platelet distribution width may increase diagnostic yield, although these parameters were not collected in the present study [25].

### Optimal test combinations

The combination of the CBC at T0 and CRP at T24 had the highest negative predictive value (93%) with a minimal number of tests. Considering every blood drawn represents a significant volume loss for premature neonates and carries an inherent infection risk, the use of a less invasive, but still accurate sepsis work-up strategy is worth considering. Since the majority of clinicians include a CBC in the initial workup of suspected late-onset sepsis [7], it is unlikely that an approach solely based on serial CRP measure would be adopted. Also, two CRP values drawn at T0 and T24 had a non-superior negative predictive value than the combined CBC at T0 and CRP at T24. Further, there were no additional benefit to repeat any blood tests at T48. Lastly, if diagnostic accuracy at the time of initial evaluation (T0) is a priority, then the combination of the CBC and CRP at T0 allows the highest sensitivity and might assist in clinical decision-making.

### Early cessation of antibiotics

An important benefit of using reliable laboratory markers is to help in the decision to discontinue empirical antimicrobial. Recent studies reported a 97% negative predictive value of blood culture at 36 h [26, 27]. The high negative predictive value of negative blood cultures at 36 h, combined with the 93% negative predictive value of the two-step approach described above (CBC at T0 and CRP at T24) could be used together to support discontinuation of antimicrobials at that time rather than waiting the traditional 48-h time point. This, in turn, would help mitigate drug-associated adverse events, decrease the likelihood of selection of resistant organisms and Candida sp. [26], necrotizing enterocolitis and potential hearing impairment [28], in addition to reducing healthcare costs [6]. Additionally, discontinuing antibiotics after 36 h reduces blood draws required for antibiotic dosing when using aminoglycosides and/or vancomycin are used as empiric therapy.

### Variations in CRP, white blood cell count and I/T ratio

The analysis of ROC curves associated with the absolute change from T0 to T24 of the CRP, the WBC count and the I/T ratio has clinical implications. For example, it improves the WBC count relevance compared to a single value at T0. These results reinforce the importance of a marker’s kinetic rather than its absolute value at a specific timing. However, when trying to minimize the amount of blood tests, one should consider that the AUC of the change in white blood cell count from T0 to T24 was not significantly different than a single CRP done at T24. Consequently, repeating a CBC to monitor the white blood cell count variation is not warranted. Furthermore, as there is no current positivity cut-off for the absolute increase in white blood cell count, its interpretation is subject to variability.

### Strengths and limitations

An important strength of this study is the single cohort of VLBW infants. The retrospective design and inclusion of all newborns with a late-onset sepsis evaluation may have led to the inclusion of patients with other conditions associated with an increased CRP. Indeed, among infants with false positive CRP results at T24, there were 8 postoperative infants and 14 who were treated for necrotizing enterocolitis, two conditions known to increase CRP values [2, 10, 29]. Also, infants with a diagnosis of ventilator-associated pneumonia and elevated CRP were not considered as having a late-onset sepsis since the objective of the study was to assess diagnostic accuracy in predicting culture-proven bloodstream infections or meningitis. Also, there were no standardized criteria for diagnosis of ventilator-associated pneumonia in VLBW infants on the NICU during the study period. We did not collect data on vaccination and intraventricular hemorrhage which might also have increased false positive rates [10]. All these conditions, by increasing the CRP false-positive results may have contributed to underestimating its specificity. However, the inclusion of these patients would have had little effect on the negative predictive value. Finally even though missing data were imputed using LOCF method, their proportion varied between 5% and 33% for the different lab tests and time points. To ensure that this did not bias the results, sensitivity analysis without imputation were carried and showed identical conclusions.

## Conclusion

In summary, this study is the first that describes the combined CRP and CBC for the diagnosis of late-onset sepsis in a large cohort of VLBW neonates. Our results emphasize that suspected late-onset sepsis initial workup should include both CRP and CBC if the decision to start antibiotics is uncertain. Also, it supports early antibiotics cessation after 36 h of negative cultures if the combined CBC at T0 and CRP at T24 are negative. Using a two tests combination strategy could reduce iatrogenic consequences of late-onset sepsis investigations in VLBW infants. However, further studies are required to determine if different cut-off values of CRP at different timings during late-onset sepsis evaluation in VLBW infants could increase sensitivity.

## Abbreviations

AUC: Area under the curve
CBC: Complete blood count
CoNS: Coagulase-negative *staphylococci*
CRP: C-reactive protein
GEE: Generalized estimating eqs.
I/T: Immature neutrophil/total neutrophil
LOCF: Last observation carried forward
ROC: Receiver operating characteristics
VLBW: Very low birth weight
